# Impact of prior flavivirus immunity on Zika virus infection in rhesus macaques

**DOI:** 10.1371/journal.ppat.1006487

**Published:** 2017-08-03

**Authors:** Michael K. McCracken, Gregory D. Gromowski, Heather L. Friberg, Xiaoxu Lin, Peter Abbink, Rafael De La Barrera, Kenneth H. Eckles, Lindsey S. Garver, Michael Boyd, David Jetton, Dan H. Barouch, Matthew C. Wise, Bridget S. Lewis, Jeffrey R. Currier, Kayvon Modjarrad, Mark Milazzo, Michelle Liu, Anna B. Mullins, J. Robert Putnak, Nelson L. Michael, Richard G. Jarman, Stephen J. Thomas

**Affiliations:** 1 Viral Diseases Branch, Walter Reed Army Institute of Research, Silver Spring, Maryland, United States of America; 2 Center for Virology and Vaccine Research, Beth Israel Deaconess Medical Center, Harvard Medical School, Boston, Massachusetts, United States of America; 3 Pilot Bioproduction Facility, Walter Reed Army Institute of Research, Silver Spring, Maryland, United States of America; 4 Entomology Branch, Walter Reed Army Institute of Research, Silver Spring, Maryland, United States of America; 5 Veterinary Services Program, Walter Reed Army Institute of Research, Silver Spring, Maryland, United States of America; 6 Henry M. Jackson Foundation, Bethesda, Maryland, United States of America; 7 Military HIV Research Program, Walter Reed Army Institute of Research, Silver Spring, Maryland, United States of America; 8 Walter Reed Army Institute of Research, Silver Spring, Maryland, United States of America; University of North Carolina at Chapel Hill, UNITED STATES

## Abstract

Studies have demonstrated cross-reactivity of anti-dengue virus (DENV) antibodies in human sera against Zika virus (ZIKV), promoting increased ZIKV infection *in vitro*. However, the correlation between *in vitro* and *in vivo* findings is not well characterized. Thus, we evaluated the impact of heterotypic flavivirus immunity on ZIKV titers in biofluids of rhesus macaques. Animals previously infected (≥420 days) with DENV2, DENV4, or yellow fever virus were compared to flavivirus-naïve animals following infection with a Brazilian ZIKV strain. Sera from DENV-immune macaques demonstrated cross-reactivity with ZIKV by antibody-binding and neutralization assays prior to ZIKV infection, and promoted increased ZIKV infection in cell culture assays. Despite these findings, no significant differences between flavivirus-naïve and immune animals were observed in viral titers, neutralizing antibody levels, or immune cell kinetics following ZIKV infection. These results indicate that prior infection with heterologous flaviviruses neither conferred protection nor increased observed ZIKV titers in this non-human primate ZIKV infection model.

## Author summary

Zika virus (ZIKV) is a mosquito-borne, emerging flavivirus. In addition to asymptomatic or mild febrile manifestations, ZIKV infection in humans is associated with comparatively rare congenital and neurological abnormalities. Previous in vitro studies have demonstrated cross-reactivity of antibodies derived from dengue virus infections with ZIKV, as well as antibody-mediated enhancement of infection in cell culture. Therefore, there is concern among vaccinologists and the public health community that the more severe disease manifestations could result from prior immunity, either from natural infection or vaccination, to antigenically-related flaviviruses (e.g., dengue, yellow fever, Japanese encephalitis). In this study, we evaluated the impact of prior immunity to dengue or yellow fever virus on ZIKV infection in a non-human primate model. After confirming that our immune cohorts were capable of recapitulating the published in vitro observations of cross-reactivity and enhancement, we looked for alteration of viral titers in peripheral blood, urine, cerebrospinal fluid, saliva, and vaginal secretions, as well as alterations in gross pathology, clinical parameters, and immune response, as compared to our flavivirus-naïve cohort. Importantly, no significant differences between flavivirus-naïve and primed animals were observed in viral titers or biofluid distribution, neutralizing antibody levels, or immune cell kinetics following ZIKV infection.

## Introduction

Zika virus (ZIKV), is a flavivirus originally isolated from a sentinel rhesus macaque in the Zika forest of Uganda in 1947 [1] and a human in Nigeria in 1953 [2]. Transmission occurs primarily in a human-mosquito cycle, though ZIKV can also be transmitted sexually and from mother to fetus [3]. ZIKV transmission expanded eastward from Africa to Asia where other flaviviruses are endemic, including Japanese encephalitis virus (JEV) and the four dengue virus (DENV) serotypes [4, 5]. ZIKV reached the Americas in 2015, a region with co-circulating DENV1-4, YFV vaccination programs, and risk of natural YFV infection [4, 6]. The epidemic intensified in Brazil and spread, with autochthonous transmission documented in fifty-seven other countries, including the United States [7, 8].

ZIKV infection is typically asymptomatic or manifests as a mild clinical syndrome that often includes fever, rash, and arthralgia, and may also include conjunctivitis, pruritus, muscle pain, headache, and malaise [9]. ZIKV infection has been associated with a range of congenital and neurological abnormalities to include fetal microcephaly, with an estimated risk of 0.88–13.2% following first trimester infections [10], and Guillain-Barre syndrome (GBS) in approximately 1 in 4,000 infected children and adults [11]. To date, 21 countries and territories are reporting either an increased incidence of GBS or laboratory confirmation of ZIKV among GBS cases [7].

Numerous ZIKV vaccine candidates are in pre-clinical and early clinical development to include safety studies planned in flavivirus primed populations [12–15]. Assessing the safety, immunogenicity, and potential for clinical benefit of ZIKV vaccine candidates in flavivirus-naïve populations would be more straightforward but the current epidemiology of ZIKV transmission makes this scenario unlikely. Heterologous flavivirus priming of vaccine trial volunteers could impact the safety profile of, and immune response to, candidate ZIKV vaccines. Pre-existing immunity to non-ZIKV flaviviruses from natural infection or immunization may also provide some level of cross-protection or attenuation upon subsequent ZIKV exposure.

Concerns that ZIKV infections may be exacerbated by pre-existing flavivirus immunity are largely driven by epidemiologic and in vitro observations of DENV infections. Cross-reactive but non- or poorly-neutralizing antibodies generated against the precursor membrane (prM) or envelope (E) structural proteins of one DENV serotype are thought to mediate enhancement of infection upon subsequent exposure to a heterologous serotype through a process termed antibody-dependent enhancement (ADE). In ADE, virions in complex with these antibodies gain an additional means to infect FcγR-bearing cells, leading to increased virus replication. Accordingly, studies have observed increasing viral loads in association with subsequent disease presentation in humans. [16–20]. Cell mediated immunity has also been proposed as a contributor to enhanced infection and disease [21]. Despite these observations, epidemiologic findings regarding increased disease severity during heterologous flavivirus infections have been inconsistent. In a prospective cohort, the presence of JEV-neutralizing antibodies was associated with an increased incidence of symptomatic primary DENV infections [22], while a second study found a reduction in the severity of dengue disease in a JE-vaccinated cohort [23]. Importantly, immune sera or antibodies to DENV and YFV have demonstrated cross-reactivity with ZIKV and the capability to promote increased infection in vitro [24–28].

In the present study we investigated the potential for prior flavivirus immunity to alter ZIKV titers and immune responses using rhesus macaques, an accepted model of flavivirus infection, that were previously infected with DENV2, DENV4, or YFV as could reasonably be seen in a flavivirus endemic population encountering ZIKV for the first time. Viral load, presence of ZIKV in numerous biofluids, clinical events following infection, immune cell phenotypes, and serologic responses were compared between flavivirus-immune and -naïve animals. This study seeks to address the recently published in vitro findings of cross-reactive antibodies and the potential to promote increased ZIKV titers in the context of sequential, in vivo flavivirus infections.

## Results

### Study design

Study events and specimen collections are outlined in S1 Table. Individual animal information is outlined in S2 Table. Six and five rhesus macaques were infected with DENV and YFV, respectively, ≥420 days prior to subcutaneous inoculation with ZIKV (Brazil-ZKV2015). Specific time intervals and neutralizing antibody titers from these prior infections are outlined in S2 and S3 Tables. Fourteen additional animals confirmed to be serologically naïve for JEV, YFV, West Nile virus (WNV), DENV serotypes 1–4, and ZIKV prior to study start were inoculated with ZIKV contemporaneously. As such, the three experimental groups are termed naïve, DENV-immune, and YFV-immune. One animal from the DENV- or YFV-immune groups and one animal from the serologically naïve group were sacrificed in sex-matched pairs at intervals throughout the study for future investigations of ZIKV tissue distribution and pathology. Sacrificing of animals at different time points throughout the study limited the availability of biological samples at later time points, accounting for the disparity in data set sample sizes between earlier and later time points. Samples from all remaining animals were included in all analyses unless otherwise stated.

### Pre-existing immunity and in vitro assessment

**Fig 1:**
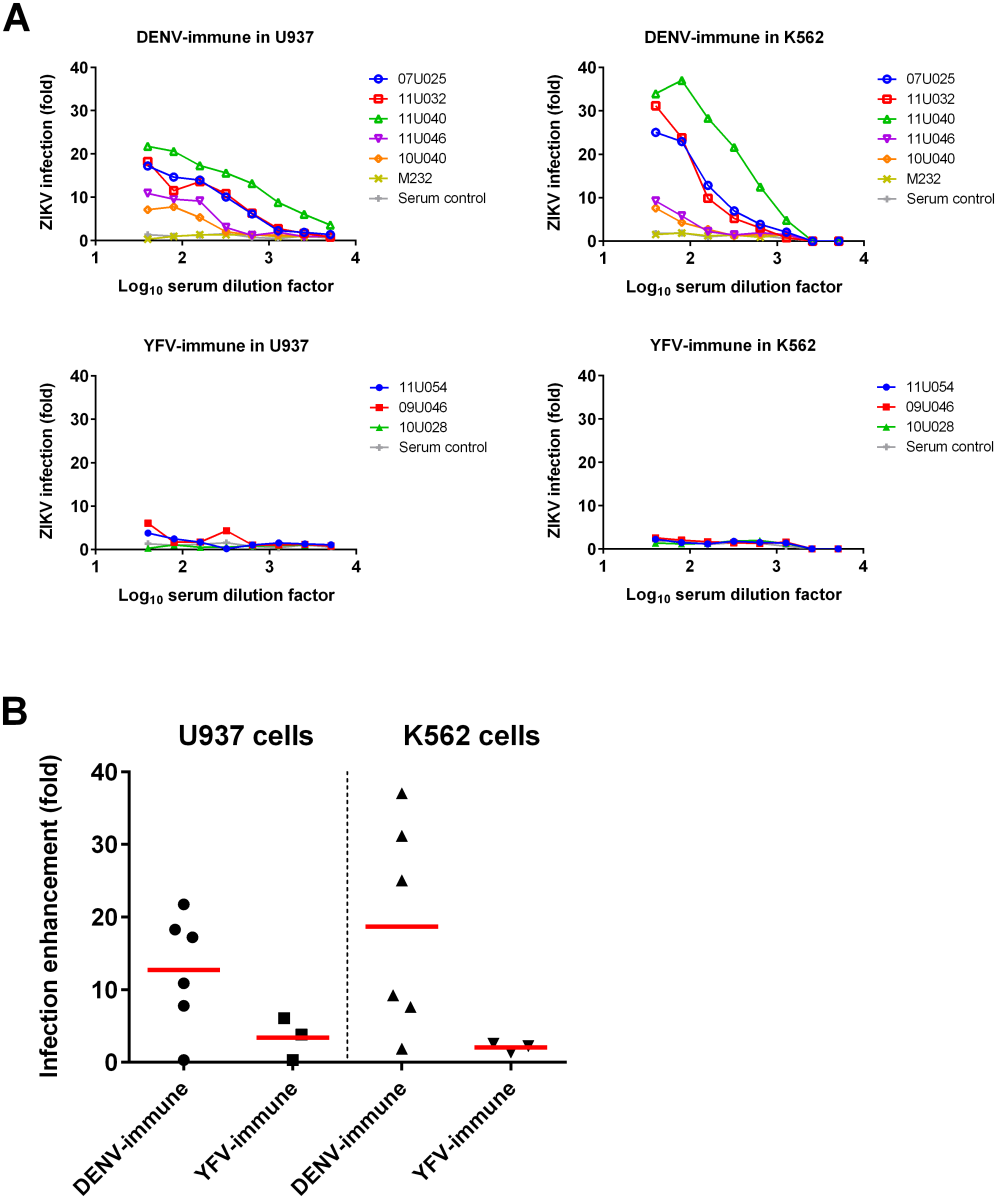
Antibody-dependent enhancement of ZIKV infection in vitro for day 0 sera. Antibody-dependent enhancement of infection of U937 and K562 cells by ZIKV was assessed after 24 hours using the flow cytometry based infection assay. (A) Fold-enhancement curves for individual animals. Means of duplicate values are displayed. (B) Peak fold-enhancement values with the mean indicated by a red bar. Serum control is from naïve group animal 10U043. DENV-immune and YFV-immune sample sizes (n) for this analysis are 5 and 3, respectively.

To verify that the sera of the DENV- and YFV-immune groups were capable of promoting increased ZIKV infection in vitro, as has been demonstrated in other studies, day 0 sera were assessed. End-point ELISA binding curves demonstrated moderate amounts of cross-reactive antibody to ZIKV in the DENV-immune group and much less cross-reactive antibody in the YFV-immune group (S1 Fig). Day 0 DENV-immune sera also appreciably cross-neutralized ZIKV, with FlowNT50 titers ranging from 40–582. However, the neutralization curves indicate less potent NAbs and incomplete neutralization of virus infectivity compared to anti-ZIKV control sera (S2 Fig). Finally, the capability of sera from the DENV-immune group and the three most cross-reactive YFV-immune animals (by end-point ELISA) to promote increased ZIKV infection was measured using a flow cytometry-based assay of infection in FcγR-bearing cells (U937 & K562). Pre-incubation of ZIKV with DENV-immune sera promoted infection with 12.7- and 18.7-fold higher mean peak detected values in U937 and K562 cells, respectively, than virus incubated with naïve control sera (Fig 1). As expected, when YFV-immune sera were tested, these poorly cross-reactive sera promoted comparatively lower ZIKV infection, with mean peak detected values of 3.4- and 2.0-fold higher in U937 and K562 cells, respectively, compared to naïve control sera.

### ZIKV replication and distribution

**Fig 2:**
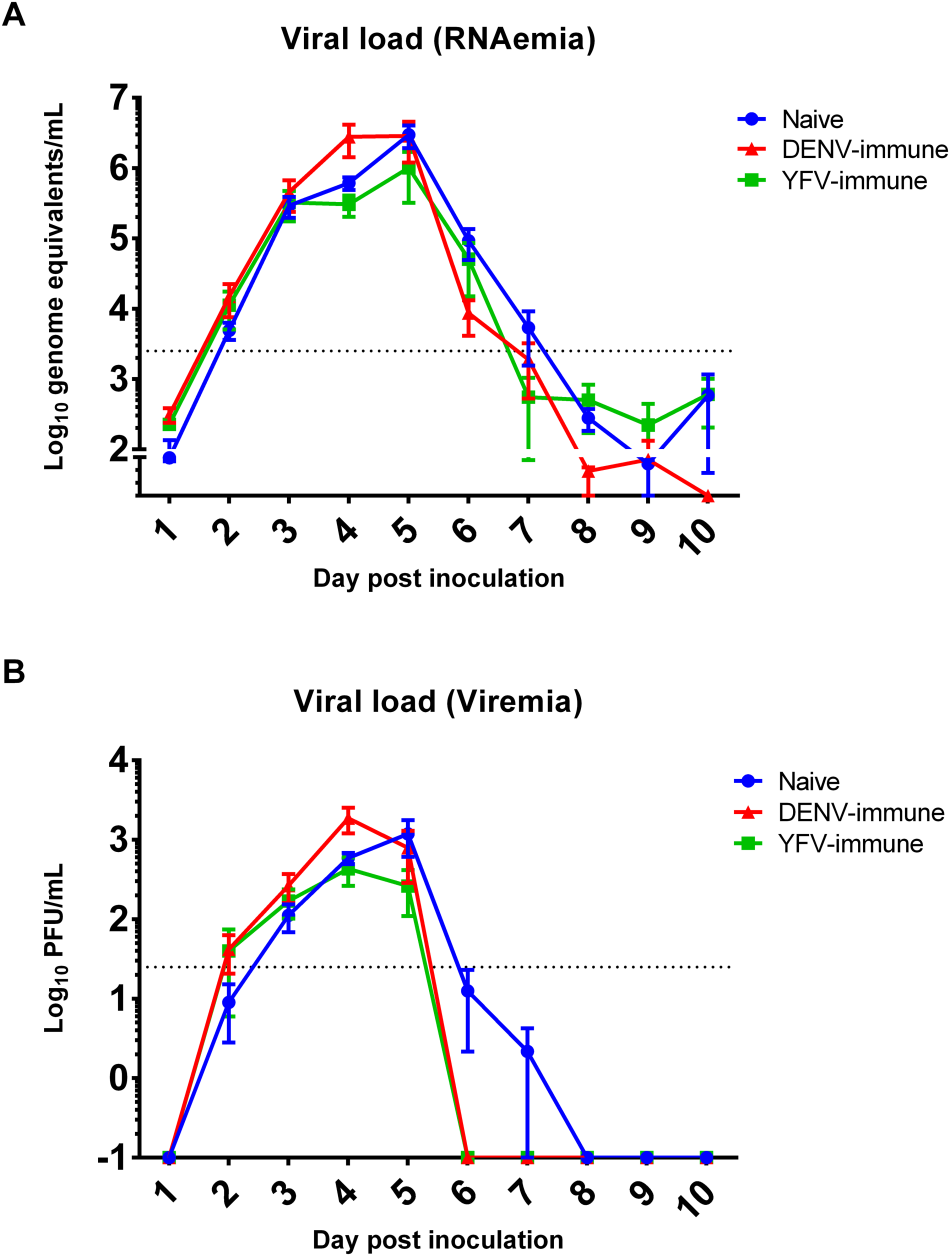
ZIKV titers in peripheral blood. ZIKV viral load was determined using RT-qPCR (A, RNAemia) and Vero cell plaque assay (B, Viremia). Dotted lines indicate the limit of detection (RNAemia = 2500, Viremia = 25). Serum specimens were used for days 1, 3, 5, 6, 8, and 10. Plasma specimens were used for days 2, 4, 7, and 9. Naïve group days 6 and 7 values represent two and one positive specimen, respectively. Means with SEM are displayed.

In order to determine the impact of prior flavivirus infection on ZIKV titers and biofluid distribution in vivo, and in light of the increased ZIKV infection observed in vitro, we quantified ZIKV viral load in the peripheral blood, urine, cerebrospinal fluid (CSF), vaginal fluid and saliva. These biofluids represent many of those most relevant to current ZIKV detection assays, as well as to ZIKV transmission, including vector-borne, sexual, and nonsexual human-to-human transmission, and to ZIKV-associated neurological sequelae [3, 7, 29]. No significant differences in RNAemia (p = 0.52, [2,23] degrees of freedom [d.f.], F statistic = 0.67) or viremia (p = 0.76, [2,23] d.f., F statistic = 0.28) were observed between the three experimental groups (Fig 2), nor were there differences observed in the respective day of peak titer (p = 0.45; p = 0.08), magnitude of peak titer (p = 0.12; p = 0.10), or duration of RNAemia or viremia (p = 0.60; p = 0.76). The group by time interaction in the mixed model was significant for RNAemia (p = 0.0084, [18,161] d.f., F statistic = 2.09) but not for viremia (p = 0.34, [18,161] d.f., F statistic = 1.12); post-hoc analysis of RNAemia by day post infection indicated that the source of this significance was isolated to days 1, 8, and 10. It should be noted that all day 1 values, and most of days 8 and 10 values, were below the limit of detection (10 genome equivalents per reaction) and days 8 and 10 demonstrated a comparatively limited number of positive samples (nine and four total, respectively), suggesting low biological significance of this finding. The relationship between RNAemia and viremia is shown by individual animals in S3 Fig, and coincides well when considering the respective limits of detection and the ratio of genome equivalents to plaque forming units (PFU) determined previously (approx. 1000:1 [12]). All biofluid types tested exhibited ZIKV-positive specimens at one or more time points in all groups with the exception of vaginal fluid samples from DENV-immune animals, though this group contained only 2 females on day 0. No association was observed between the magnitude or proportion of ZIKV-positive biofluids and experimental group (Table 1, S4 Fig).

**Table 1:** Distribution of ZIKV in biofluids determined by RT-qPCR.

Specimen	DPI	Naïve					DENV-immune					YFV-immune				
		Pos.	Neg.	Eq.	SNC	Total	Pos.	Neg.	Eq.	SNC	Total	Pos.	Neg.	Eq.	SNC	Total
**Urine**	**Day 4**	2	9	0	2	13	1	3	0	1	5	0	5	0	0	5
	**Day 7**	2	8	0	2	12	2	2	0	1	5	0	2	1	1	4
	**Day 10**	3	7	0	0	10	2	2	0	0	4	1	2	0	0	3
	**Day 14**	1	6	1	1	9	0	4	0	0	4	0	2	0	0	2
	**Day 22**	1	6	0	0	7	1	2	0	0	3	0	1	0	0	1
**CSF**	**Day 4**	5	8	0	0	13	2	3	0	0	5	1	4	0	0	5
	**Day 7**	3	7	2	0	12	3	1	1	0	5	2	1	1	0	4
	**Day 10**	2	8	0	0	10	2	1	0	1	4	1	2	0	0	3
	**Day 14**	2	5	2	0	9	2	2	0	0	4	0	1	0	1	2
	**Day 22**	1	5	1	0	7	1	2	0	0	3	0	1	0	0	1
**Vaginal fluid**	**Day 4**	0	8	0	0	8	0	2	0	0	2	1	3	0	0	4
	**Day 7**	3	4	0	0	7	0	2	0	0	2	2	1	0	0	3
	**Day 10**	1	4	1	0	6	0	1	0	0	1	2	1	0	0	3
	**Day 14**	0	4	1	0	5	0	0	1	0	1	0	2	0	0	2
	**Day 22**	0	4	0	0	4	0	1	0	0	1	0	1	0	0	1
**Saliva**	**Day 4**	0	13	0	0	13	0	5	0	0	5	0	5	0	0	5
	**Day 7**	9	3	0	0	12	4	0	1	0	5	2	2	0	0	4
	**Day 10**	1	8	1	0	10	3	1	0	0	4	0	3	0	0	3
	**Day 14**	0	9	0	0	9	0	4	0	0	4	0	1	1	0	2
	**Day 22**	0	7	0	0	7	0	3	0	0	3	0	1	0	0	1

The declining sample size (n) for each group in the Total column reflects sacrifices beginning on day 2 post infection.

Pos. = positive value (detected value within the range of the standard curve), Neg. = negative value (undetected value), Eq. = equivocal value (detected value below the range of the standard curve), SNC = specimen not collected, Total = sum of all Pos., Neg., Eq., & SNC specimens

### Kinetics of immune response to ZIKV

**Fig 3:**
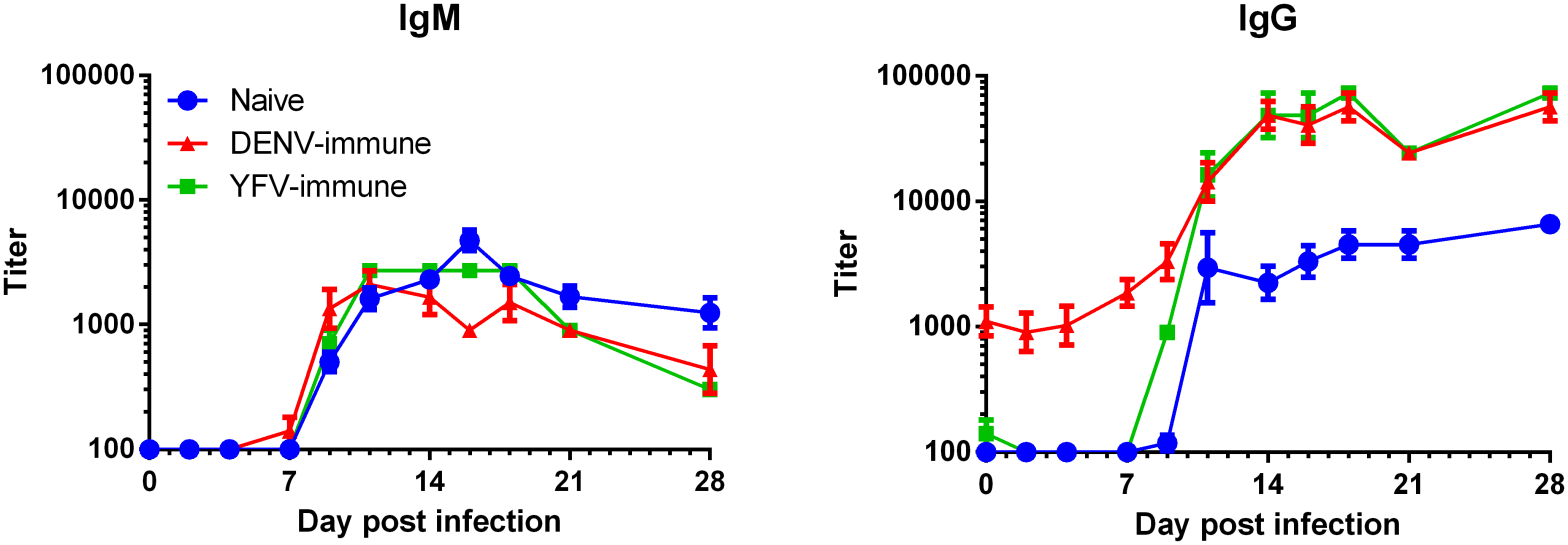
ZIKV IgM and IgG kinetics. Anti-ZIKV IgM and IgG kinetics were determined by ZIKV-capture ELISA for each of the exposure groups. The flavivirus naïve group has a characteristic primary flavivirus antibody response with detectable levels of ZIKV-reactive IgM preceding IgG, and both isotypes plateauing at a similar level. By contrast the DENV- and YFV-immune groups demonstrate characteristic secondary flavivirus IgM and IgG kinetics with IgM following similar kinetics to a primary infection and IgG kinetics reaching a substantially higher peak titer than seen in primary ZIKV infection. Notably, the DENV-immune animals had substantial levels of ZIKV-reactive IgG on day 0, whereas YFV-immune animals had much less. Means with SEM are displayed.

We next sought to determine if prior flavivirus infection could alter the development of immune responses against ZIKV. Anti-ZIKV IgM and IgG kinetics measured by ZIKV-capture ELISA demonstrated classic exposure responses in all experimental groups (Fig 3). IgM titers rose between days 7 and 9, plateaued within two weeks, and began to decline by day 28. Although IgM titers in the DENV-immune and YFV-immune animals appeared to decline moderately faster than in the naïve animals, the overall IgM titer curves did not differ significantly between groups (p = 0.26, [20,133] d.f., F statistic = 1.21). In contrast, IgG titer curves differed significantly between groups throughout the duration of the study (p<0.0001, [20,133] d.f., F statistic = 7.43). IgG in the DENV-immune group cross-reacted with ZIKV from day 0 at a mean titer of 1100, rose around day 7, and plateaued by day 14 lasting through the last time point tested (day 28). IgG titers in the YFV-immune group did not appreciably bind ZIKV on day 0, but displayed nearly identical development to that of the DENV-immune group, achieving similar maximum titer and duration. IgG titers in the naïve group displayed similar kinetics to that of the immune groups, but plateaued approximately 13-fold lower. The peak IgG titers were significantly different between groups (p = 0.029, 2 d.f., F statistic = 4.19). IgM and IgG kinetics are displayed by individual animal in S5 Fig.

**Fig 4:**
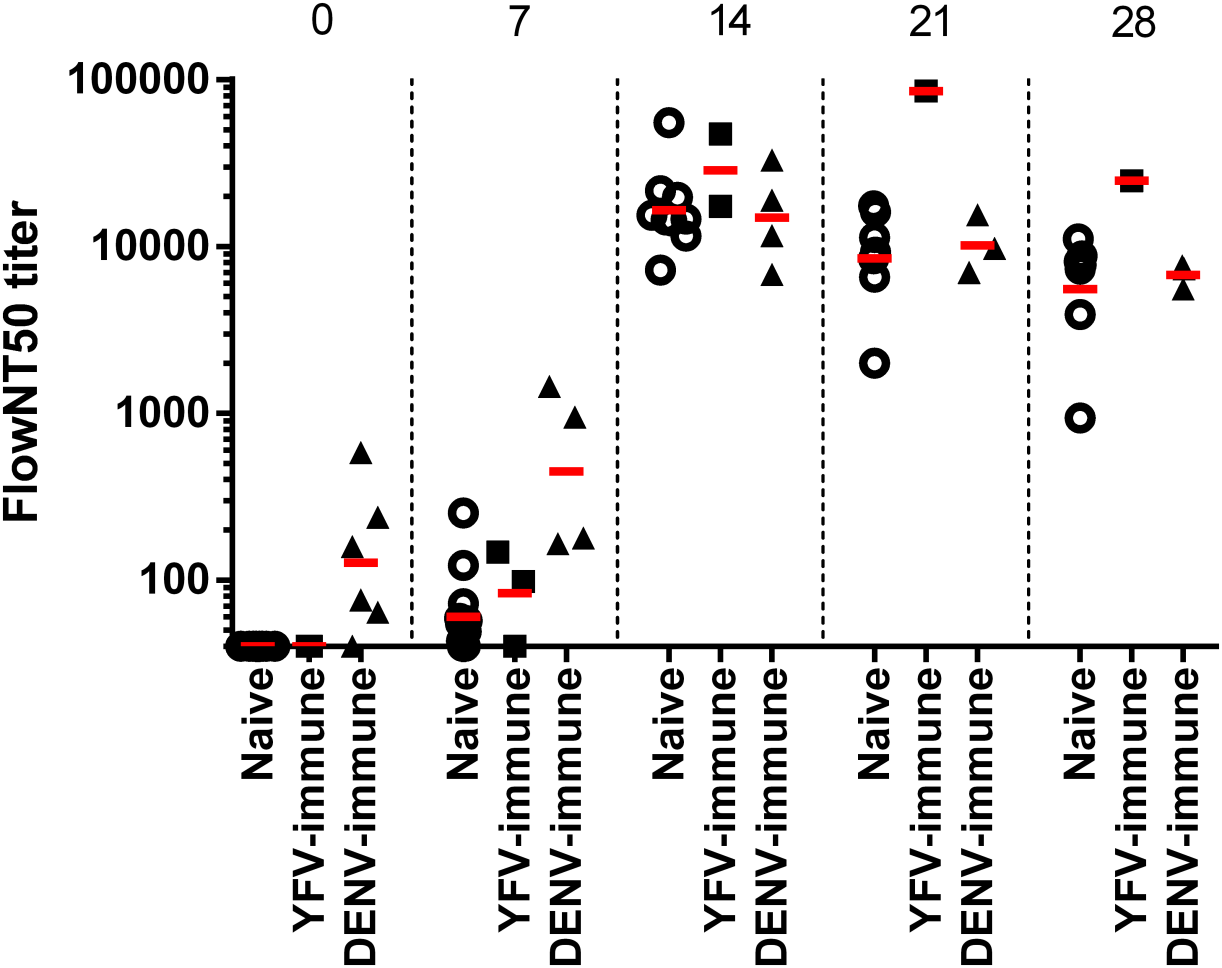
Kinetics of neutralizing antibody. Zika virus flow cytometry-based assay 50% neutralization (FlowNT50) titers were determined on days 0, 7, 14, 21, and 28 post-infection. The FlowNT50 titers for individual animals within each group are shown with the geometric mean titer (GMT) indicated with a red bar. The respective sample sizes (n) for naïve, YFV-immune, and DENV-immune groups were 14, 5, and 6 on day 0; 12, 3, and 4 on day 7; 9, 2, and 4 on day 14; 7, 1, and 3 on day 21; 7, 1, and 3 on day 28. The GMTs for all groups begin to rise on day 7, peak to a similar level on day 14 and remain high out to day 28. The single YFV-immune animal remaining on day 21 and 28 of the study demonstrated a moderately higher FlowNT50 titer compared to animals in the naïve and DENV-immune groups.

Neutralizing antibody (NAb) kinetics against ZIKV of sera from naïve, DENV-immune, and YFV-immune animals were also assessed. Pre-infection (day 0) neutralization titers are displayed in S4 Table; only the DENV-immune animals demonstrated cross-reactive, anti-ZIKV NAb titers, in agreement with the ELISA binding results. Despite this expected difference in neutralization capacity, NAb kinetics following ZIKV inoculation did not differ among groups (Fig 4). In all experimental groups, mean anti-ZIKV NAb titers rose from day 7, peaked by day 14 at similar titers among groups, and remained high out to the last time point measured (day 28).

**Fig 5:**
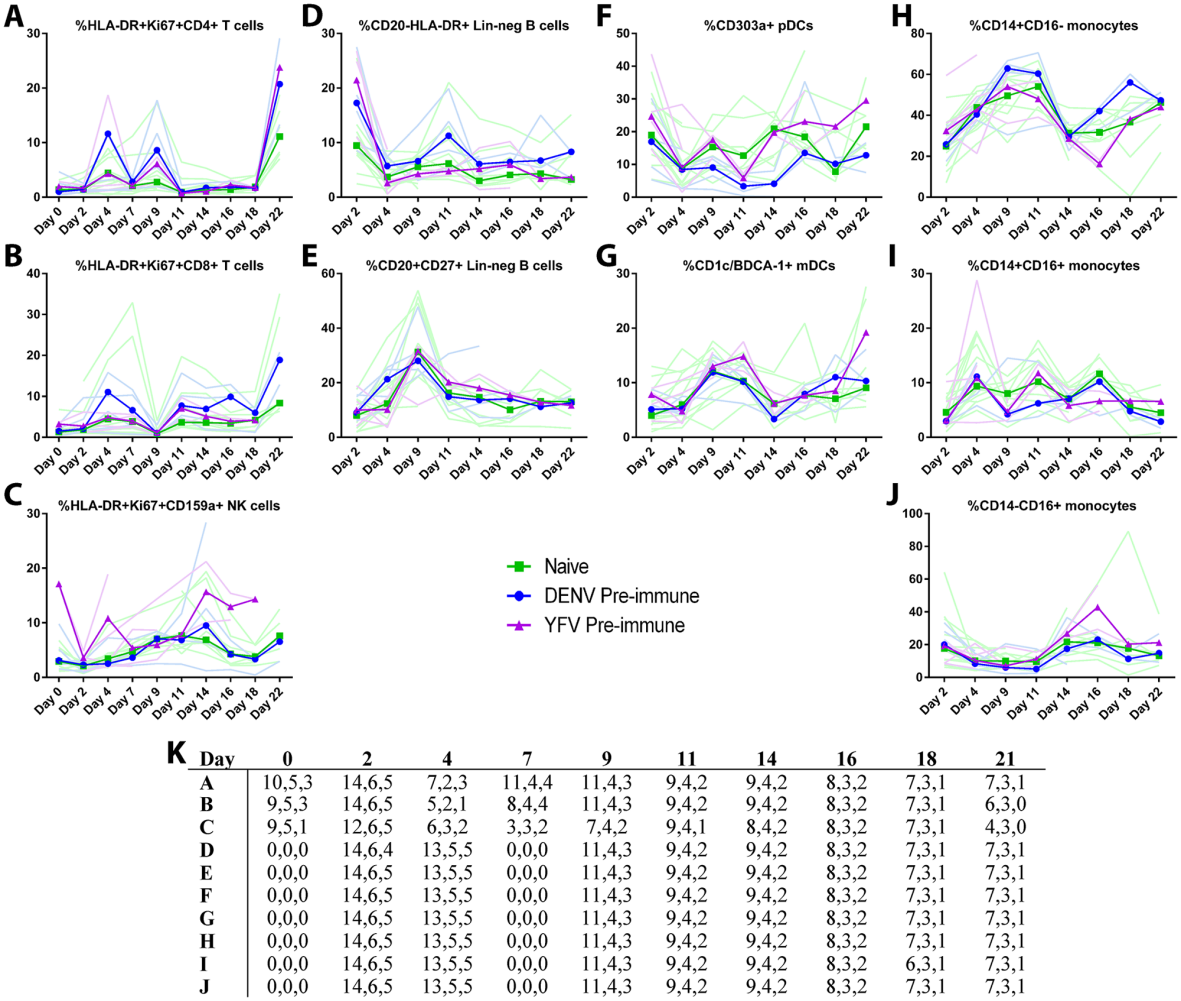
Cell-mediated immune activation during acute ZIKV infection. Ex vivo whole blood immunophenotyping was performed during acute ZIKV infection in rhesus macaques. Multiparametric flow cytometry was used to identify various immune cell subsets, including (**A**) activated CD4^+^ and (**B**) CD8^+^ T cells; (**C**) activated CD159a^+^ NK cells; (**D**) CD20^-^HLA-DR^+^ and (**E**) CD20^+^CD27^+^ Lineage-negative B cells (CD3^-^CD14^-^CD16^-^CD11c^-^); (**F**) CD303a^+^ pDCs and (**G**) CD1c/BDCA-1^+^ mDCs (HLA-DR^+^CD20^-^CD14^-^CD16^-^); and (**H**) CD14^+^CD16^**-**^, (**I**) CD14^+^CD16^+^, and (**J**) CD14^-^CD16^+^ monocytes (HLA-DR^+^CD20^-^). Shown are individual monkeys (faint lines) as well as medians (bold lines) of naïve (green), DENV-immune (blue), and YFV-immune (purple) groups. Day 0 is the day of infection. (**K**) Table displays the sample size (n) that met inclusion criteria for each group (Naïve, DENV-immune, YFV-immune) at each time point for each cell subset A-J.

Potential alterations to the immune response were assessed further by characterizing the kinetics of cellular immune activation via ex vivo whole blood immunophenotyping. Multiparametric flow cytometry was performed every 2–3 days through day 22 to identify activated CD4^+^ and CD8^+^ T cells and natural killer cells (CD159a^+^), as determined by HLA-DR and Ki67 dual expression, as well as CD20^-^HLA-DR^+^ and CD20^+^CD27^+^ lineage-negative B cells (CD3^-^CD14^-^CD16^-^CD11c^-^), plasmacytoid (CD303a^+^) and myeloid-lineage (CD1c/BDCA-1^+^) dendritic cells (HLA-DR^+^CD20^-^CD14^-^CD16^-^), and three populations of monocytes (HLA-DR^+^CD20^-^) defined by their CD14 and CD16 expression profiles (CD14^+^CD16^-^, CD14^+^CD16^+^, and CD14^-^CD16^+^). Gating strategies are displayed in S6 Fig. Importantly, no statistically significant differences in median frequencies were found between groups for any cell population on any individual day. With regard to the kinetics of cellular immune activation irrespective of group, as expected, we saw evidence of T and NK cell activation within the first two weeks post-infection as well as movement in the frequencies of various B cell, DC, and monocyte populations (Fig 5). Activated CD4^+^ T cells displayed two peaks, days 4 and 9, as did activated CD8^+^ T cells, with the second peak delayed relative to CD4+ T cells. Activation of both CD4^+^ and CD8^+^ T cells increased at day 22 consistent with the peak adaptive response occurring after the clearance of viremia as seen previously for DENV in humans [30–32]; investigation of later time points are necessary to further define the kinetics of this response. Plasmacytoid dendritic cells (pDCs) decreased in frequency in the first two weeks post-infection, whereas mDCs and CD14^+^CD16^-^ monocytes increased. CD20^+^CD27^+^ lineage-negative B cells, expected to contain the memory B cell population, peaked in frequency on day 9; this kinetic profile is more consistent with what we might expect for plasmablasts, necessitating future experiments demonstrating ex vivo production and/or specificity of antibody to further define this population.

### Clinical course, clinical laboratory, and pathologic findings

Following virus challenge, all animals were observed daily for clinical course, clinical laboratory, and, in the sacrificed animals, gross pathologic evidence of ZIKV disease. Detailed findings are presented in S1 Text. There were no local or systemic signs of disease noted in the naïve compared to the primed animal groups. The same was true across a broad range of hematologic and biochemical values. Cerebrospinal fluid analyses also failed to reveal a difference between groups. Finally, except for mild peripheral lymphadenopathy noted in both naïve and YFV-primed animals, there were no other findings on gross pathologic examination.

## Discussion

Our results indicate that pre-existing immunity to antigenically related flaviviruses neither increased nor diminished the magnitude of ZIKV viral titers and did not alter ZIKV kinetics in peripheral blood and other biofluids, nor did it alter the kinetics of ZIKV-induced immune responses, relative to flavivirus-naïves in our cohort of rhesus macaques. Further, although there were minor fluctuations in serum chemistry values, no clinical signs were evident in the animals in association with experimental group. Experimental groups with prior flavivirus infection exhibited significantly higher peak anti-ZIKV IgG titers, but this difference did not translate into differences in peak antibody neutralization capacity. In contrast to the ZIKV infection data presented here, secondary DENV infections have revealed highly serotype-cross-reactive CD4+ and CD8+ T cells, which have been hypothesized to contribute to disease severity [30, 31, 33–35]. Future experiments will address the specificity and/or cross-reactivity of these cells, although T-cell responses have been shown to be predominately specific between ZIKV and DENV upon ex vivo stimulation [24].

The present study benefits from utilizing animals that were previously infected with antigenically related flaviviruses 14 months or more prior to inoculation with ZIKV. This places the animals well past the cross-protective interval and ensures a mature immune response as described for DENV [36–38]. In order to confirm that the animals used in this study were capable of generating the same in vitro biological activities as previously described, we performed ELISAs, neutralization titrations, and antibody-mediated infection assays similar to those previously published [24–28]. Accordingly, our DENV-immune animals demonstrated the capability to promote substantially increased levels of ZIKV infection in both of the human cell lines used previously, U937 and K562, and at similar starting dilutions. Despite this in vitro capability, no alterations to ZIKV viral titers were apparent in vivo in the specimens tested. These data are consistent with a previous study of DENV infection in human infants [39], as well as with studies of flaviviruses for which clinically apparent ADE is not known to occur; WNV, for example, exhibits in vitro ADE when using sub-neutralizing concentrations of antibody, but this does not appear to translate to observable enhancement of human illness [40]. Similarly, another study demonstrated no effects on ZIKV viremia or tissue distribution when an anti-DENV mAb with known in vitro infection-mediating capability was administered to a non-permissive mouse model 24 hours prior to ZIKV inoculation, though the authors acknowledge this discrepancy could be due to a limitation of the mouse model [24]. Additional studies with a shorter interval between initial flavivirus priming or infection and subsequent ZIKV challenge, as well as studies including pregnant females, would be required to more fully examine the potential impact of heterologous flavivirus priming.

Rhesus macaques have successfully demonstrated immunologically-induced increases of DENV titers in peripheral blood previously, with both passive transfer of antibody and sequential infection experiments capable of invoking both humoral and cellular immune effects. Rhesus macaques sequentially infected with DENV2 following a heterologous serotype of DENV successfully produced consistently higher daily DENV2 viremia titers as compared to primary DENV2 infection [41]. Intravenous passive transfer with sub-neutralizing concentrations of anti-DENV pooled human cord-blood serum 15 minutes prior to infection promoted up to a 51-fold increase in cumulative DENV2 viremia titers [42]. More recently, a >100-fold increase in DENV4 viremia titers was promoted in rhesus macaques by administration of sub-neutralizing concentrations of a humanized, chimpanzee-derived, anti-DENV monoclonal antibody 24 hours prior to infection [43].

Analysis of the data from this and other investigations supports the use of non-human primates as a reproducible and informative ZIKV infection model [44]. Comprehensive autopsy and organ system examinations, currently in progress, will provide additional information in this regard. However, similar to DENV, limitations exist with using NHPs as a ZIKV disease model. This is especially apparent for relatively rare flavivirus disease manifestations, such as dengue hemorrhagic fever or accelerated disease or neurologic syndromes seen with ZIKV, where the requirement to study a hundred or more animals is infeasible. Alternatively, immunologic enhancement of ZIKV infection and any potentially associated clinical manifestations might not occur in NHPs in vivo.

Our experiments do not definitively rule out a possible role of immunologic enhancement in ZIKV-associated neurological and congenital abnormalities; the present data do not address antibody-virus complexes in the context of traversing anatomical barriers, perhaps through utilization of the Fc-receptor-bearing Hofbauer cells of the placenta, nor does the present study incorporate pregnant NHPs whose varying immunological states might affect the outcome [45, 46]. As animal models of maternal-fetal ZIKV transmission improve and more is known regarding the pathophysiology of ZIKV and in utero infection, targeted studies in flavivirus primed, pregnant animals can be performed. That said, the in vitro experiments in this and previous studies have demonstrated the potential for anti-DENV antibodies to promote subsequent ZIKV infection of FcγR-bearing cell lines in vitro, but the inability of this phenomenon to translate to increased ZIKV titers in the biofluids of an otherwise relevant in vivo model is of key importance in order to inform the approach to large scale clinical studies in flavivirus endemic human populations. It is only in these types of clinical studies where the impact of previous flavivirus infection on subsequent ZIKV diseases and its impact on the safety and efficacy of ZIKV vaccines can be definitively established.

## Methods

### Ethics statement

This study was approved by the Institutional Animal Care and Use Committee, and research was conducted in compliance with the Animal Welfare Act and other federal statutes and regulations relating to animals. Experiments involving animals adhered to principles stated in the *Guide for the Care and Use of Laboratory Animals* from the National Research Council. The Walter Reed Army Institute of Research (WRAIR) is fully accredited by the Association for Assessment and Accreditation of Laboratory Animal Care International.

### Study design

Previous studies have demonstrated cross-reactivity of anti- DENV antibodies in human sera against ZIKV, promoting increased ZIKV infection of cells *in vitro*. However, the correlation between *in vitro* and *in vivo* findings is not well characterized. As such, we evaluated the impact of heterotypic flavivirus immunity on ZIKV titers in biofluids of rhesus macaques utilizing flaviviruses with geographic and antigenic similarity: DENV serotypes 2 and 4, and yellow fever virus. One animal from the DENV- or YFV-immune groups and one animal from the serologically naïve group were sacrificed in sex-matched pairs at intervals throughout the study for future investigations of ZIKV tissue distribution and pathology. Sample size was determined based upon availability of previously infected animals, rather than by sample size estimation using a desired power or known effect size. The number of naïve animals was chosen to exceed the combined total of previously infected animals such that future analysis of long-term serological data would still contain five animals through the final necropsy. All animals were infected with ZIKV contemporaneously in a controlled laboratory experiment. Study events and specimen collections were determined prior to study start and are outlined in S1 Table. Individual animal information, time intervals since prior flavivirus exposure, and prior exposure neutralizing antibody titers are outlined in S2 and S3 Tables. All samples were included in all analyses except where described below. Group allocation and data analysis occurred in an unblinded manner.

### Animals

Healthy, adult, Indian origin rhesus macaques (*Macaca mulatta*) were obtained from Covance Research Products, Alice, Texas (colony bred). All animals utilized in this study were tested for JEV, WNV, YFV, and DENV1-4 antibodies by a sensitive screening virus neutralization assay prior to initial infection. Animals were anesthetized with ketamine/acepromazine (11 mg /Kg) / (0.55 mg/Kg), intramuscularly (IM), with a 1 cc syringe and 21 to 23 gauge, 3/4 to 1 inch needle in the caudal thigh prior to all procedures. Euthanasia was humanely performed via exsanguination in accordance with the AVMA Guidelines on Euthanasia prior to euthanasia after deep sedation with ketamine (5–12 mg/Kg) and dexmedetomidine (0.04 mg/kg). Following blood collection, death was ensured by administration of sodium pentobarbital IV 1.0ml/4.5kg (86.4mg/kg) via catheter. Death was confirmed by physical exam and auscultation to verify cardiac arrest by a licensed veterinarian.

### Cell lines

U937 (ATCC no. CRL-1593.2, ATCC, Manassas, VA), K562 (ATCC no. CCL-243, ATCC), and U937-DC-SIGN (ATCC no. CRL-3253, ATCC) cell lines were utilized in this study. These lines were verified to be authentic, using short tandem repeat profiling, morphology, and cytochrome C oxidase I testing, and free of contamination by ATCC prior to use.

### Viruses

ZIKV strain Brazil ZKV2015 (ZIKV-BR) was inoculated subcutaneously as 6.0 log_10_ genome equivalents (3.0 log_10_ PFU) diluted in unsupplemented RPMI media to one mL. ZIKV stock was generated as previously described [12]. DENV2 strain S16803, DENV4 strain H241, and YFV strain 17D were produced in Vero-81 cell culture and inoculated subcutaneously at a dose of 4.5 log_10_ PFU in 0.5 ml of sterile culture fluid. DENV1-4 (strain Western Pacific 1974, S16803, CH53489, TVP-360, respectively), YFV strain 17D, ZIKV strain Paraiba-Brazil/2015, and JEV strain SA14-14-2 were utilized in ELISA, MN50, FlowNT50, and PRNT assays. Viruses utilized in MN50 and PRNT assays were produced in Vero cells. Viruses utilized in ELISA and FlowNT50 assay were produced in C6/36 cells. Virus used in ELISA was purified by ultracentrifugation over a 30% sucrose solution and resuspended in PBS.

### Mucosal swabbing, vaginal swabbing, and urine processing

Samples were collected on days 4, 7, 10, 14, and 22. Saliva and vaginal fluid were collected using sterile, dry cotton swabs then placed into 1 mL of EMEM supplemented with 10% fetal bovine serum, 1% sodium bicarbonate, and 1% L-glutamine. Urine was collected via cystocentesis under anesthetization. Samples were aliquoted and stored at -80°C until use.

### RT-qPCR

Viral load in sera or plasma (RNAemia), CSF, urine, saliva, and vaginal fluid were determined using an RT-qPCR assay as previously described [13]. RNA was extracted from sera on days 1, 3, 5, 6, 8, and 10 and from plasma on days 2, 4, 7, and 9 due to animal welfare collection volume constraints. Viral loads were calculated as genome equivalents/mL. Limit of detection herein is defined as the lowest value in the standard curve that can be detected in greater than 95% of assay runs, which was 10 genome equivalents/reaction or 2500 genome equivalents/mL.

### Plaque titration

Plaque titration of viruses in inocula and ZIKV viral load (viremia) in sera were determined by standard plaque assay on Vero cell monolayers. Limit of detection was 25 plaque-forming units/mL.

### ELISA

End-point anti-ZIKV IgG and IgM ELISA titers on days 0, 2, 4, 7, 9, 11, 14, 16, 18, 22, and 28 were determined using ZIKV-capture ELISA as previously described [47]. This protocol utilized negative control, pre-infection serum from animal 10U015, positive control anti-ZIKV serum from animal 09U024 on day 16, and goat anti-monkey IgM HRP-conjugated secondary antibody (catalog no. 074-11-031, KPL, Gaithersburg, MD) or goat anti-monkey IgG (H+L) HRP-conjugated secondary antibody (catalog no. PA1-84631, ThermoFisher Scientific, Waltham, MA); the anti-IgG antibody might react with additional antibody isotypes due to the use of light chains in the immunogen preparation, though this has not been determined empirically.

### MN50

Neutralizing antibody titers in heat-inactivated sera preceding (day -420) and 28 days post-inoculation (day -392) with DENV or YFV were determined using a 96-well, high-throughput, ELISA-based microneutralization assay (MN50) in Vero cells as previously described [48].

### FlowNT50

Neutralizing antibody titers in heat-inactivated sera pre-infection (days -30 and 0) and post-inoculation with ZIKV (days 7, 14, 21, 28) were determined using a 96-well, high-throughput, flow cytometry-based neutralization assay cells as previously described, with modification [49]. Serial dilutions of sera are mixed with an equal volume of virus, diluted to achieve 10–15% infection of cells/well, and incubated for 1 hr at 37°C. After 1 hr of incubation, an equal volume of medium (RPMI-1640 supplemented with 10% FBS, 1% penicillin/streptomycin, 1% L-glutamine (200mM), and 1% non-essential amino acids (10mM)) containing 5x10^4^ U937-DC-SIGN cells are added to each serum-virus mixture and incubated 18–20 hr overnight in a 37°C, 5% CO2, humidified incubator. Following overnight incubation, the cells are fixed, permeabilized and immunostained with flavivirus group-reactive mouse monoclonal antibody 4G2, and secondary polyclonal goat anti-mouse IgG PE-conjugated antibody (catalog no. 550589, BD Biosciences, San Jose, CA). The percent infected cells are quantified on a BD Accuri C6 Plus flow cytometer (BD Biosciences, San Jose, CA). Data were analyzed by nonlinear regression to determine 50% endpoint titers in GraphPad Prism 6. Day 7 serum samples for animals 07U025 and 09U024 were excluded from analysis due to insufficient volume.

### PRNT

Standard plaque-reduction neutralization tests (PRNT) on Vero cell monolayers were utilized as a screening tool of pre-study sera for determining serologically flavivirus-naïve animals at a 1:10 dilution of heat-inactivated sera. Neutralizing antibody titers in pre-infection (day -30) sera against YFV were determined by 50% end-point titration (PRNT50).

### Antibody-dependent enhancement assay

In vitro antibody-dependent enhancement of infection was quantified as previously described, with modification similar to that used in the FlowNT50 [24–26]. Beginning at 1:40, two-fold serial dilutions of heat-inactivated day 0 sera were incubated with virus (sufficient to infect 10–15% of U937-DC-SIGN cells) at 1:1 for 1 hr at 37°C. This mixture is then added to a 96-well plate containing 5x10^4^ cells (U937 or K562) per well in duplicate. Cells were infected 18–20 hr overnight in a 37°C, 5% CO2, humidified incubator. Processing and quantification continued as in the FlowNT50 methods. Fold-infection relative to control serum is reported. Samples for animals 09U024 and M228 from the YFV-immune group were not tested, as they exhibited minimal binding by ELISA.

### Isolation of plasma and PBMC from whole blood

Whole blood samples were drawn on days 0, 2, 4, 7, 9, 11, 14, 16, 18, and 22 post-infection from all live animals (in-life draws) and from all necropsied animals on the day of sacrifice (necropsy draws). All blood processing was performed at room temperature unless otherwise specified. For in-life draws, one 2.7-mL blood collection tube containing sodium citrate (BD-363083, BD Biosciences) was drawn from each animal. Once received in the lab, the blood was immediately transferred to a 15-mL conical tube and 100 μL was transferred into each of three FACS tubes for ex vivo phenotyping analysis. The remaining blood was centrifuged at 300 x*g* for 8 min, and approximately 700 μL plasma was transferred to a 2-mL purple-cap tube for subsequent centrifugation (1200 x*g* for 8 min), aliquoting, and cryopreservation at -80°C. The blood was then resuspended in approximately 3 mL of warm phosphate-buffered saline (PBS) and layered on top of 3 mL ficoll (Ficoll-Paque PLUS, GE Healthcare) before centrifugation at 400 x*g* for 30 min. The PBMC layer was transferred to a clean 15-mL conical tube and washed several times in PBS. PBMC were then resuspended in a solution of 90% fetal bovine serum (FBS) plus 10% dimethyl sulfoxide (DMSO) and transferred into cryovials for subsequent freezing. Samples were cryopreserved using a StrataCooler (Agilent Technologies, Santa Clara, CA), placed at -80°C for 24–72 hours followed by long-term storage in vapor-phase liquid nitrogen. For necropsy draws, several 8-mL cell preparation tubes containing sodium citrate (BD362761, BD Biosciences) were drawn from each sacrificed animal. Once received in the lab, the blood was immediately transferred to a sterile bottle and diluted 1:1 in warm PBS, then transferred onto a layer of ficoll at a 2:1 ratio in 50-mL conical tubes. Tubes were centrifuged at 400 x*g* for 30 min, and the PBMC layer was isolated and transferred to a clean 50-mL tube for subsequent washing and cryopreservation as described above for the in-life samples.

### Ex vivo whole blood immunophenotyping

Whole blood samples were collected on days 0, 2, 4, 7, 9, 11, 14, 16, 18, and 22 for ex vivo immunophenotyping by multiparametric flow cytometry. Blood was collected in blood collection tubes containing sodium citrate and transferred to FACS tubes for staining with three different antibody panels, including one intracellular staining panel (Panel 1) and two surface-only panels (Panels 2 and 3). Panel 1 included the following antibodies: anti-CD3-A700 (clone SP34-2, BD Biosciences, San Jose, CA); anti-CD4-BV711 (clone SK3, BD Biosciences); anti-CD8-BV510 (clone SK1, BD Biosciences); anti-CD45-BV785 (clone D058-1283, BD Biosciences); anti-CD159a-PE-Cy7 (clone Z199, Beckman Coulter, Indianapolis, IN); anti-HLA-DR-BV650 (clone L243, BioLegend, San Diego, CA); and anti-Ki67-A488 (clone B56, BD Biosciences). Panel 2 included the following antibodies: anti-CD3-APC-Cy7 (clone SP34-2, BD Biosciences); anti-CD11c-BV421 (clone 3.9, BD Biosciences); anti-CD14-A700 (clone M5E2, BD Biosciences); anti-CD16-BV510 (clone 3G8, BD Biosciences); anti-CD20-FITC (clone L27, BD Biosciences); anti-CD27-BV650 (clone O323, BioLegend); and anti-HLA-DR-PE-Dazzle594 (clone L243, BioLegend). Panel 3 included the following antibodies: anti-CD16-APC-Cy7 (clone 3G8, BioLegend); anti-CD1c-BV421 (clone L161, BioLegend); anti-CD11c-PE-Dazzle594 (clone 3.9, BioLegend); anti-CD14-A488 (clone M5E2, BD Biosciences); anti-CD303a-APC (clone 201A, eBioscience, San Diego, CA); and anti-HLA-DR-BV605 (clone L243, BioLegend). For surface staining, 100 μL staining mix was added directly to 100 μL whole blood and incubated at room temperature (RT) for 15 min. Then 1 mL per tube red blood cell lysis buffer (BD Pharm Lyse, BD Biosciences) was added, and the tubes were vortexed prior to incubation at RT for an additional 15 min. Samples were then washed several times in flow wash buffer (2% fetal bovine serum in phosphate-buffered saline). For Panels 2 and 3, the samples were fixed using 4% formaldehyde (RT, 15 min) and washed. For Panel 1, samples were resuspended in a fixation/permeabilization buffer (Foxp3 Staining Buffer Set, eBioscience) for 15 min at RT followed by washing and an additional permeabilization step for 15 min at RT. Samples were stained intracellularly with Ki67 antibody diluted in perm buffer for 15 min at RT and then washed. Data were acquired on a BD LSRFortessa and analyzed using FlowJo v10 and GraphPad Prism v6 software. Data points with less than 1000 events in the parent population (for example, total CD4+ T cells) were excluded from the analysis. Panel 2 and 3 data from Days 0 and 7 were excluded from the analysis due to technical issues.

### Pathology

Complete necropsies were performed under biosafety level 2 by a veterinary pathologist immediately following euthanasia at scheduled post-exposure time points per protocol. Tissues from all major organ systems were collected from each animal and immersion fixed in 10% neutral buffered formalin. Select fresh tissues were collected for future RNA and virus isolation investigations. In addition, select tissues were identified for future transmission electron microscopy and fixed in a solution of 4% formaldehyde with 1% glutaraldehyde buffered by sodium phosphate monobasic at pH 7.4. Histopathology samples from all major organ systems were routinely processed, embedded in paraffin, sectioned, and stained with hematoxylin and eosin.

### Clinical chemistry and hematology

Hematologic analysis was obtained from whole blood samples collected in purple-topped EDTA tubes. Hematologic analysis was conducted using a Sysmex XT-2000iV Hematology Analyzer (Sysmex America, Lincolnshire, IL). The hematology parameters analyzed included white blood cell (WBC) count, red blood cell (RBC) count, hemoglobin (HGB), percentage hematocrit (HCT), mean corpuscular volume (MCV), mean corpuscular hemoglobin (MCH), mean cell hemoglobin concentration (MCHC), platelet (PLT) count, red cell distribution width (RDW), mean platelet volume (MPV), reticulocyte percentage, and reticulocyte count. References ranges utilized were in-house intervals based upon routine physicals of 200 rhesus monkeys from the WRAIR animal colony not involved in studies. Serum chemistry analysis was obtained from whole blood collected in gold-topped serum separator tubes. Serum was analyzed for glucose, urea nitrogen, creatinine, sodium, potassium, chloride, carbon dioxide, calcium, phosphorus, cholesterol, triglycerides, total protein, albumin, aspartate aminotransferase (AST), alanine transaminase (ALT), lactate dehydrogenase (LDH), creatine kinase (CK), alkaline phosphatase (ALKP), gamma glutamyltransferase (GGT), and total bilirubin using a Vitros 350 Chemistry System (Ortho Clinical Diagnostics, Raritan, NJ).

### Cerebrospinal fluid analysis

Cerebral spinal fluid was collected via lumbar puncture (in-life and time of sacrifice) or cisternal puncture (time of sacrifice). Tubes were placed into 4°C upon collection and manipulated on ice. Tubes were mixed using low-speed vortex. Cell counts were performed using trypan blue (0.4%) viability staining on a hemocytometer. For in-life (low volume) collections, aliquots of neat CSF were frozen at -80°C for protein and virus quantification. For collections performed at the time of sacrifice, a neat aliquot was frozen at -80°C for protein quantification. The remaining volume was centrifuged at 800xg for 5 minutes at 4°C. The supernatant was stored in aliquots at -80°C for virus quantification. The remaining cell pellet was resuspended in approximately 500uL residual CSF and mixed 1:1 with fetal bovine serum containing 20% dimethyl sulfoxide. Tubes were placed into StrataCoolers (Agilent Technologies, Santa Clara, CA), frozen at -80°C for 24 hours, then transferred to LN_2_ for later use. CSF total protein analysis was obtained from CSF aliquots using a Vitros 350 Chemistry System (Ortho Clinical Diagnostics, Raritan, NJ).

### Statistical analyses

Differences in proportions of abnormal laboratory values were assessed by Fisher’s exact test and adjusted for multiple comparisons using the False Discovery Rate (FDR) correction. Changes from baseline laboratory values were assessed by the Kruskal-Wallis test and were adjusted using FDR. Differences in clinical parameters (weight and temperature) and changes from baseline values were also assessed by the Kruskal-Wallis test and were also adjusted using FDR. Mean magnitude of peak titers were compared across groups using the Kruskal-Wallis test. Day of peak RNAemia or viremia was assessed by Kaplan-Meier analysis and logrank test. Viral loads were log_10_-transformed to meet test assumptions and changes in viral load and antibody titers were examined using methods of longitudinal data analysis. Specifically, we estimated changes within individual macaques and between experimental and comparison groups from a random effects linear model using the SAS MIXED procedure for repeated measures. This method was chosen to account for unequal and declining sample sizes, and it includes an estimate of variation within group. Model-selection strategies were employed to adjust for potentially important covariates. Variance-covariance structure and model-fit diagnostics were assessed for each model. Differences in peak antibody binding titers were assessed using an ANOVA model. Differences in cellular phenotyping data were determined using the Kruskal-Wallis test and adjusted using FDR. All data analyses were performed using SAS version 9.4 (SAS Institute, Cary, North Carolina). All tests performed were two-tailed tests unless otherwise noted. Significance was assessed at α = 0.05.

### Data availability

The datasets generated and analyzed during the current study are available from the corresponding author on reasonable request.

## Supporting information

S1 Fig ELISA binding curves of day 0 sera. DENV-immune sera demonstrated more cross-reactivity with ZIKV than YFV-immune sera prior to infection. Serum control is from the naïve group animal 10U015. AU = absorbance units. Means with SEM are displayed.
(TIF)

S2 Fig ZIKV neutralization curves of day 0 sera. DENV-immune sera cross-neutralized ZIKV with lower potency than anti-ZIKV control serum with the curves having shallower slopes and incomplete neutralization of virus infectivity by comparison. Serum control is from naïve group animal 10U043. Anti-ZIKV serum is a known positive human serum. Dotted lines at 50 and 10 represent the 50% and 90% neutralization, respectively. Means with non-linear regression curves are displayed.
(TIF)

S3 Fig Individual animal ZIKV titers in peripheral blood. ZIKV viral load was determined using RT-qPCR (RNAemia, blue, right axis) and Vero cell plaque assay (viremia, red, left axis). Dotted lines indicate the limit of detection (RNAemia = 2500 genome equivalents per mL (blue), viremia = 25 PFU per mL (red)). Serum specimens were used for days 1, 3, 5, 6, 8, and 10. Plasma specimens were used for days 2, 4, 7, and 9. RNAemia and viremia curves are overlaid for each animal.
(TIF)

S4 Fig Distribution of ZIKV in bodily fluids by individual animal. ZIKV viral load in peripheral blood, saliva swab, vaginal swab, CSF, and urine specimens was determined using RT-qPCR. These curves are overlaid for each animal. Dotted lines indicate the limit of detection (2500 genome equivalents per mL). CSF values at sacrifice time points were obtained from centrifuged (cell-free) CSF, rather than unprocessed CSF.
(TIF)

S5 Fig Individual ZIKV IgM and IgG kinetics. Anti-ZIKV IgM and IgG kinetics were determined by ZIKV-capture ELISA. IgM and IgG titer curves are overlaid by individual animal.
(TIF)

S6 Fig Gating strategies to identify immune cell subsets by flow cytometry. Ex vivo immunophenotyping was performed on whole blood samples acquired during acute ZIKV infection in rhesus macaques. Three multiparametric flow cytometry staining panels were used to identify various immune cell subsets. (A) Panel 1 was specific for T and NK cells, which were first defined by their low FSC-A and SSC-A profiles, followed by singlet gating and expression of CD45. T cells were further defined as CD3+ and either CD4+ or CD8+. NK cells were defined as CD3-CD159a+. All three subsets were assessed for activation via dual expression of HLA-DR and Ki67. (B) Panel 2 identified B cells by low FSC-A and SSC-A, singlet gating, and lack of expression of CD14, CD3, CD16 and CD11c. Two B cell subsets were identified, including CD20-HLA-DR+ cells and CD20+CD27+ cells. (C) Panel 3 was designed to identify monocytes and dendritic cells (DCs) by way of high FSC-A and SSC-A profiles, singlet gating, and expression of HLA-DR with lack of CD20. Three subsets of monocytes were defined by their CD14 and CD16 expression profiles (CD14+CD16-, CD14+CD16+, and CD14-CD16+). Two populations of DCs were defined, CD14-CD16-CD303a+ (plasmacytoid DCs) and CD14-CD16-CD1c+ (also known as BDCA-1; myeloid-lineage DCs).
(TIF)

S7 Fig Clinical measurements: Weight, temperature, and serum chemistries. Weight and temperature were recorded at all study time points and are displayed as individual animals. Serum chemistries were performed on study days 0, 7, and 22. Dotted lines represent the upper and lower bounds of the references range. Means with SEM are displayed.
(TIF)

S8 Fig Clinical measurements: Complete blood counts. Complete blood counts were performed on study days 0, 7, and 22. Dotted lines represent the upper and lower bounds of the references range. Means with SEM are displayed.
(TIF)

S9 Fig Total protein quantity in the CSF. The normal concentration of total protein in the cerebrospinal fluid for rhesus macaques is 8–50 mg/100 ml (or mg/dL), represented by the dotted lines. Data is displayed by individual animal.
(TIF)

S10 Fig Infiltrating cell counts in the CSF. Total cell counts in cerebrospinal fluid were performed using Trypan blue viability staining on a hemocytometer. Data is displayed by individual animal. Data points in red had known RBC content.
(TIF)

S1 Table Study design. (DOCX)

S2 Table Individual rhesus macaque information. (DOCX)

S3 Table Neutralization titers pre- and post-DENV or YFV infection of immune group rhesus macaques. (DOCX)

S4 Table Pre-infection neutralizing antibody titers. (DOCX)

S1 Text Detailed clinical course, clinical laboratory, and pathologic findings. (DOCX)
