# Supplementary figures and images for: Impact of prior flavivirus immunity on Zika virus infection in rhesus macaques
Source: PLoS Pathog. 2017 Aug 3;13(8):e1006487. doi: 10.1371/journal.ppat.1006487 (PMC5542404; doi:10.1371/journal.ppat.1006487)

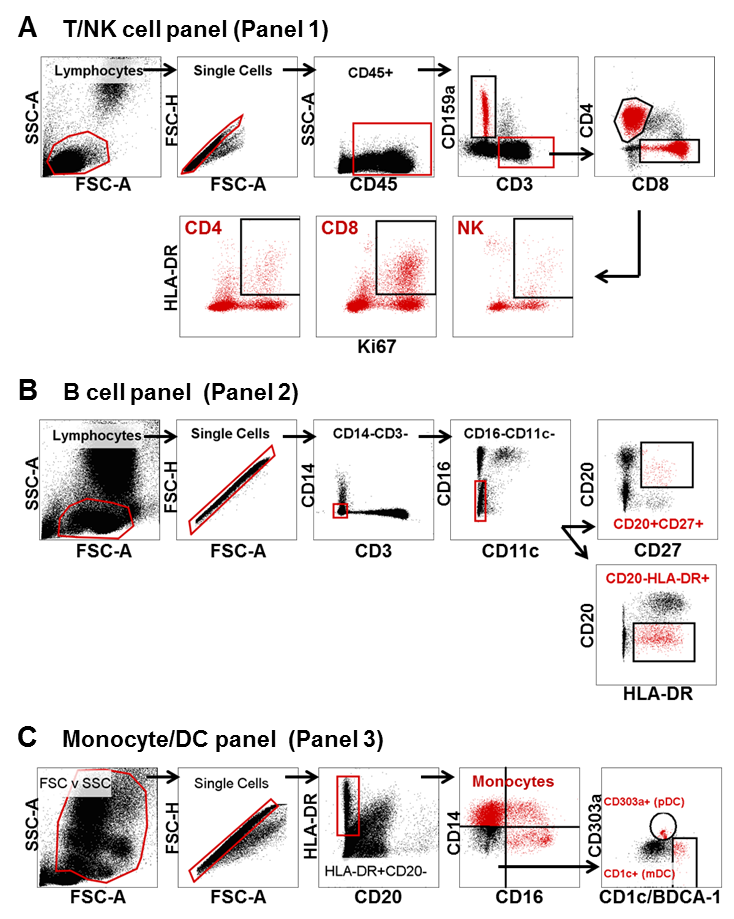

Supplement: S6 Fig — Ex vivo immunophenotyping was performed on whole blood samples acquired during acute ZIKV infection in rhesus macaques. Three multiparametric flow cytometry staining panels were used to identify various immune cell subsets. (A) Panel 1 was specific for T and NK cells, which were first defined by their low FSC-A and SSC-A profiles, followed by singlet gating and expression of CD45. T cells were further defined as CD3+ and either CD4+ or CD8+. NK cells were defined as CD3-CD159a+. All three subsets were assessed for activation via dual expression of HLA-DR and Ki67. (B) Panel 2 identified B cells by low FSC-A and SSC-A, singlet gating, and lack of expression of CD14, CD3, CD16 and CD11c. Two B cell subsets were identified, including CD20-HLA-DR+ cells and CD20+CD27+ cells. (C) Panel 3 was designed to identify monocytes and dendritic cells (DCs) by way of high FSC-A and SSC-A profiles, singlet gating, and expression of HLA-DR with lack of CD20. Three subsets of monocytes were defined by their CD14 and CD16 expression profiles (CD14+CD16-, CD14+CD16+, and CD14-CD16+). Two populations of DCs were defined, CD14-CD16-CD303a+ (plasmacytoid DCs) and CD14-CD16-CD1c+ (also known as BDCA-1; myeloid-lineage DCs). (TIF) [file ppat.1006487.s006.tif]
