# Supplementary material for: Impact of prior flavivirus immunity on Zika virus infection in rhesus macaques
Source: PLoS Pathog. 2017 Aug 3;13(8):e1006487. doi: 10.1371/journal.ppat.1006487 (PMC5542404; doi:10.1371/journal.ppat.1006487)
Supplement: S1 Table — (DOCX) [file ppat.1006487.s011.docx]

**S1 Table. Study design**

| **Event** | **Study Day** | | | | | | | | | | | | | | | | | | | | | | |
| --- | --- | --- | --- | --- | --- | --- | --- | --- | --- | --- | --- | --- | --- | --- | --- | --- | --- | --- | --- | --- | --- | --- | --- |
|  | -30 | 0 | 0+6h | 1 | 2 | 3 | 4 | 5 | 6 | 7 | 8 | 9 | 10 | 11 | 12 | 13 | 14 | 16 | 18 | 22 | 28 | 56 | 112 |
| Infection |  | X |  |  |  |  |  |  |  |  |  |  |  |  |  |  |  |  |  |  |  |  |  |
| Serology (sera) | X | X |  |  |  |  |  |  |  |  |  |  |  |  |  |  | X |  |  |  | X | X | X |
| CBC/diff (sera) |  | X |  |  |  |  |  |  |  | X |  |  |  |  |  |  |  |  |  | X |  |  |  |
| Chem-20 (sera) |  | X |  |  |  |  |  |  |  | X |  |  |  |  |  |  |  |  |  | X |  |  |  |
| Sera |  | X | X | X |  | X |  | X | X |  | X |  | X |  | X | X |  |  |  |  |  |  |  |
| Plasma / PBMC |  | X |  |  | X |  | X |  |  | X |  | X |  | X |  |  | X | X | X | X |  |  |  |
| Urine |  |  |  |  |  |  | X |  |  | X |  |  | X |  |  |  | X |  |  | X |  |  |  |
| Cerebrospinal fluid |  |  |  |  |  |  | X |  |  | X |  |  | X |  |  |  | X |  |  | X |  |  |  |
| Saliva swab |  |  |  |  |  |  | X |  |  | X |  |  | X |  |  |  | X |  |  | X |  |  |  |
| Vaginal swab |  |  |  |  |  |  | X |  |  | X |  |  | X |  |  |  | X |  |  | X |  |  |  |
| **Sacrificed Animals†** |  |  |  |  | X |  | X |  |  | X |  | X | X |  |  |  | X | X |  |  | X | X | X |
| Sex |  |  |  |  | M |  | F |  |  | M |  | F | F |  |  |  | M | F |  |  | F | M | F |
| Naïve ID |  |  |  |  | 09U038 |  | 11U018 |  |  | M236 |  | 10U039 | 09U029 |  |  |  | 10U032 | 10U001 |  |  | M230 | 10U047 | 10U021 |
| Immune ID |  |  |  |  | M232 |  | 11U054 |  |  | M228 |  | 11U040 | 09U046 |  |  |  | 07U025 | 09U024 |  |  | 10U028 | 11U046 | 11U032 |

**†**Cerebrospinal fluid, sera, and plasma/PBMC were collected from all sacrificed animals at the respective time points.

X indicates that the event occurred on the respective study day.
