# Supplementary material for: Impact of prior flavivirus immunity on Zika virus infection in rhesus macaques
Source: PLoS Pathog. 2017 Aug 3;13(8):e1006487. doi: 10.1371/journal.ppat.1006487 (PMC5542404; doi:10.1371/journal.ppat.1006487)
Supplement: S2 Table — (DOCX) [file ppat.1006487.s012.docx]

**S2 Table. Individual rhesus macaque information**

| **Animal ID** | **Sex** | **Age** | **Day 0 Weight (Kg)** | **Previous infection** |
| --- | --- | --- | --- | --- |
| 09U029 | Female | 6 | 7.02 | Naïve |
| 09U038 | Male | 6 | 8.28 | Naïve |
| 10U001 | Female | 5 | 4.94 | Naïve |
| 10U003 | Female | 5 | 6.04 | Naïve |
| 10U021 | Female | 5 | 6.16 | Naïve |
| 10U030 | Male | 5 | 7.90 | Naïve |
| 10U032 | Male | 5 | 10.40 | Naïve |
| 10U036 | Female | 5 | 4.80 | Naïve |
| 10U039 | Female | 5 | 5.34 | Naïve |
| 10U043 | Male | 5 | 7.82 | Naïve |
| 10U047 | Male | 5 | 6.80 | Naïve |
| 11U018 | Female | 4 | 4.34 | Naïve |
| M230 | Female | 6 | 8.26 | Naïve |
| M236 | Male | 5 | 11.16 | Naïve |
| 07U025 | Male | 8 | 10.32 | DENV2 (-2065 days) |
| 11U032 | Female | 4 | 6.00 | DENV2 (-420 days) |
| 11U040 | Female | 4 | 5.08 | DENV2 (-420 days) |
| 11U046 | Male | 4 | 5.60 | DENV2 (-420 days) |
| M232 | Male | 5 | 8.44 | DENV2 (-420 days) |
| 10U040 | Male | 5 | 6.86 | DENV4 (-445 days) |
| 09U024 | Female | 6 | 6.90 | YFV (-420 days) |
| 09U046 | Female | 6 | 5.88 | YFV (-420 days) |
| 10U028 | Female | 5 | 7.44 | YFV (-420 days) |
| 11U054 | Female | 4 | 5.12 | YFV (-420 days) |
| M228 | Male | 5 | 7.34 | YFV (-420 days) |
