# Supplementary material for: Impact of prior flavivirus immunity on Zika virus infection in rhesus macaques
Source: PLoS Pathog. 2017 Aug 3;13(8):e1006487. doi: 10.1371/journal.ppat.1006487 (PMC5542404; doi:10.1371/journal.ppat.1006487)
Supplement: S3 Table — (DOCX) [file ppat.1006487.s013.docx]

**S3 Table. Neutralization titers pre- and post-DENV or YFV infection of immune group rhesus macaques**

| **Animal ID** |  | **MN50 titer** | | | | | |
| --- | --- | --- | --- | --- | --- | --- | --- |
|  | **Study Day** | **JEV** | **YFV** | **DENV1** | **DENV2** | **DENV3** | **DENV4** |
| 09U024 | -420 (pre) | <10 | <10 | <10 | <10 | <10 | <10 |
| 09U024 | -392 (post) | <10 | >7290 | <10 | <10 | <10 | <10 |
| 09U046 | -420 (pre) | <10 | <10 | <10 | <10 | <10 | <10 |
| 09U046 | -392 (post) | <10 | 5880 | <10 | <10 | <10 | <10 |
| 10U028 | -420 (pre) | <10 | <10 | <10 | <10 | <10 | <10 |
| 10U028 | -392 (post) | <10 | >7290 | <10 | <10 | <10 | <10 |
| 11U032 | -420 (pre) | <10 | <10 | <10 | <10 | <10 | <10 |
| 11U032 | -392 (post) | 14 | <10 | 189 | 5513 | 82 | 293 |
| 11U040 | -420 (pre) | <10 | <10 | <10 | <10 | <10 | <10 |
| 11U040 | -392 (post) | <10 | <10 | 325 | 4940 | 27 | 333 |
| 11U046 | -420 (pre) | <10 | <10 | <10 | <10 | <10 | <10 |
| 11U046 | -392 (post) | 10 | <10 | 397 | >7290 | 291 | 1032 |
| 11U054 | -420 (pre) | <10 | <10 | <10 | <10 | <10 | <10 |
| 11U054 | -392 (post) | <10 | >7290 | <10 | <10 | <10 | <10 |
| M228 | -420 (pre) | <10 | <10 | <10 | <10 | <10 | <10 |
| M228 | -392 (post) | <10 | 1131 | <10 | <10 | <10 | <10 |
| M232 | -420 (pre) | <10 | <10 | <10 | <10 | <10 | <10 |
| M232 | -392 (post) | <10 | <10 | 38 | 1059 | <10 | 19 |

Historical titers not available for 10U040 and 07U025
