# Supplementary material for: Impact of prior flavivirus immunity on Zika virus infection in rhesus macaques
Source: PLoS Pathog. 2017 Aug 3;13(8):e1006487. doi: 10.1371/journal.ppat.1006487 (PMC5542404; doi:10.1371/journal.ppat.1006487)
Supplement: S4 Table — (DOCX) [file ppat.1006487.s014.docx]

**S4 Table. Pre-infection neutralizing antibody titers.**

| **Animal ID** | **Previous infection** | **NT_50_ titer*** | | | | | |  | **NT_90_ titer*** |
| --- | --- | --- | --- | --- | --- | --- | --- | --- | --- |
|  |  | **DENV1** | **DENV2** | **DENV3** | **DENV4** | **YFV^#^** | **ZIKV** |  | **ZIKV** |
| 07U025 | DENV2 | <40 | 544 | <40 | <40 | NT^^^ | 40 |  | <40 |
| 11U032 | DENV2 | <40 | 1286 | 49 | <40 | NT | 238 |  | <40 |
| 11U040 | DENV2 | 279 | 2146 | 48 | <40 | NT | 76 |  | <40 |
| 11U046 | DENV2 | <40 | 34211 | 108 | <40 | NT | 582 |  | <40 |
| M232 | DENV2 | <40 | 678 | <40 | <40 | NT | 64 |  | <40 |
| 10U040 | DENV4 | <40 | <40 | <40 | 962 | NT | 159 |  | <40 |
| 09U024 | YFV | NT | NT | NT | NT | 42 | <40 |  | <40 |
| 09U046 | YFV | NT | NT | NT | NT | 497 | <40 |  | <40 |
| 10U028 | YFV | NT | NT | NT | NT | 179 | <40 |  | <40 |
| 11U054 | YFV | NT | NT | NT | NT | 72 | <40 |  | <40 |
| M228 | YFV | NT | NT | NT | NT | 151 | <40 |  | <40 |

* Determined by flow cytometry-based neutralization assay in U937-DC-SIGN cells.

# Determined by PRNT_50_ in Vero cells.

^ Animals that were negative by PRNT screening (1:10 dilution) were not tested (NT) by end-point titration.
