# Supplementary material for: Impact of prior flavivirus immunity on Zika virus infection in rhesus macaques
Source: PLoS Pathog. 2017 Aug 3;13(8):e1006487. doi: 10.1371/journal.ppat.1006487 (PMC5542404; doi:10.1371/journal.ppat.1006487)
Supplement: S1 Text — (DOCX) [file ppat.1006487.s015.docx]

**S1 Text. Detailed clinical course, clinical laboratory, and pathologic findings**

Following virus challenge, all animals were observed daily for evidence of local and systemic disease such as redness or swelling at the inoculation site, dehydration, inappetence, lethargy, weight loss, self-injurious or stereotypical behaviors, and diarrhea. Two naïve group animals (10U021 and 10U036) developed mild inappetence during the course of the study, however both animals maintained body weight and additional therapy was not required. Social housing dynamics were disrupted throughout the course of the study, which likely resulted in the mild inappetence. Following CSF collection on study day 4, one naïve animal (10U039) became lethargic, which resolved within 72 hours. No additional adverse clinical effects were observed and the lethargy was not believed to be challenge related. No other abnormal clinical signs were noted in the remaining animals. Neither mean weight nor temperatures over the course of the study differed significantly between the three groups.

**Hematology and clinical chemistries.** Hematologic and clinical chemistry values did not differ significantly between groups (**S7 Fig.** and **S8 Fig.**). On day 0, one naïve (10U003) and one YFV-immune animal (10U028) exhibited slightly decreased white blood cell (WBC) counts with a slight decrease in the number of neutrophils. On day 7, 16 out of 21 remaining animals exhibited decreased WBC counts; however, nine of these animals had no concurrent reduction in differential count. Manual counts and/or microscopic examination of the blood were not performed. Two naïve (10U039, M230) and two YFV-immune animals (10U028, M228) exhibited decreased neutrophil counts on day 7. An additional two naïve (10U047, M236) and one DENV-immune animal (11U040) exhibited decreased lymphocyte counts on day 7. By day 22, only one animal (10U028) continued to have a decreased WBC count but differential counts were within reference interval. There were mild fluctuations in RBC counts and percentage hematocrits during the study. Platelet counts also exhibited mild fluctuations. One DENV-immune animal (11U032) exhibited decreased platelets on day 7 but recovered to reference interval range by day 22.

Several animals exhibited fluctuations in glucose, likely the result of epinephrine release and/or ketamine usage for sedation. Values associated with renal function (creatinine, urea nitrogen, electrolytes, total protein, and albumin) remained within reference intervals or experienced negligible fluctuations. Cholesterol and triglycerides also fluctuated during the study, likely related to the animal’s dietary status at time of blood collection. Several monkeys exhibited increases in AST (five naïve, two DENV-immune, four YFV-immune) and CK (two naïve, two DENV-immune, three YFV-immune) levels on day 7 with return to reference interval or decreased values by day 22. These fluctuations were thought to be related to muscle damage incurred by repeated sedation for sample collections. Increases in LDH were thought to be related to muscle damage or hemolysis during blood collection. On day 0, one naïve group animal (M236) exhibited at least a three-fold increase in total bilirubin; however, ALP and GGT were within normal limits making liver dysfunction less likely. This animal’s total bilirubin returned to normal by day 7. None of these fluctuations reached the threshold of statistical significance (α=0.05).

**Cerebrospinal fluid analysis**. There was no significant difference in CSF analyses between groups. The normal concentration of total protein in the cerebrospinal fluid for rhesus macaques is 8 – 50 mg/100 ml (or mg/dL) [[1](#_ENREF_1)]. Total protein exceeded this reference range starting on day 4 through day 10 for twelve animals, ranging from 51-156 mg/dL (**S9 Fig.**). On day 4, one naïve group animal (10U003) exhibited a slight increase in CSF total protein. This value returned to reference range by the next sampling on day 7. Two naïve group animals (09U029, 10U032), one DENV-immune animal (07U025), and one YFV-immune animal (10U028) exhibited increased total protein on day 7, returning to reference interval levels by the next sampling. The two animals (10U039, 11U040) sampled and sacrificed on day 9 exhibited elevated total protein. Four naïve group animals (10U030, 10U036, 10U043, 10U047) and one DENV-immune animal (10U040) sampled on day 10 exhibited elevated total protein and returned to reference interval levels by day 14. Animals within all experimental groups exhibited increased total protein in the CSF. The number of immune cells and virus titers in the CSF did not differ between the three groups, nor did these respective values correlate. There was considerable variation in total cell counts in cerebrospinal fluid among experimental groups and among individual animals within a group over the duration of the study (**S10 Fig.**). Blood contamination of CSF during collection was observed in several samples likely due to traumatic taps. Analysis of cell counts accounted for the presence of red blood cells and still showed no difference across groups.

**Gross pathology.** No gross lesions were observed in animals of any group sacrificed on days 2, 9, 14, or 16. On day 7, the flavivirus naïve animal (M236) exhibited mild enlargement of the axillary lymph nodes. On days 10 and 28, one each flavivirus naïve (09U029 and M230, respectively) and YFV-immune animal (09U046 and 10U028, respectively) exhibited mild peripheral lymphadenopathy (axillary +/- inguinal lymph node enlargement).

**References**

1. Kabat EA, Wolf A, Bezer AE, Murray JP. Studies on acute disseminated encephalomyelitis produced experimentally in rhesus monkeys. The Journal of experimental medicine. 1951;93(6):615-33. PubMed PMID: 14832406; PubMed Central PMCID: PMC2136070.
